# Strategies for optimising musculoskeletal health in the 21^st^ century

**DOI:** 10.1186/s12891-019-2510-7

**Published:** 2019-04-11

**Authors:** Rebecca Lewis, Constanza B. Gómez Álvarez, Margaret Rayman, Susan Lanham-New, Anthony Woolf, Ali Mobasheri

**Affiliations:** 1 School of Veterinary Medicine, Faculty of Health and Medical Sciences, University of Surrey, Guildford, UK; 2 School of Biosciences and Medicine, Faculty of Health and Medical Sciences, University of Surrey, Guildford, UK; 3 Centre for Sport, Exercise and Osteoarthritis Research Versus Arthritis, Queen’s Medical Centre, Nottingham, UK; 4 Department of Regenerative Medicine, State Research Institute Centre for Innovative Medicine, Vilnius, Lithuania; 5 The D-BOARD FP7 Consortium, http://www.d-board.eu; 6 The APPROACH IMI Consortium, https://www.approachproject.eu; 7 Department of Rheumatology, Royal Cornwall Hospital, Truro, UK

**Keywords:** Ageing, Musculoskeletal health, Musculoskeletal disorders, Global burden, Joint diseases, Osteoarthritis (OA), Rheumatoid arthritis (RA), Low back pain (LBP), Osteoporosis (OP), Sarcopenia, Obesity, Type II diabetes, Metabolic disease

## Abstract

We live in a world with an ever-increasing ageing population. Studying healthy ageing and reducing the socioeconomic impact of age-related diseases is a key research priority for the industrialised and developing countries, along with a better mechanistic understanding of the physiology and pathophysiology of ageing that occurs in a number of age-related musculoskeletal disorders. Arthritis and musculoskeletal disorders constitute a major cause of disability and morbidity globally and result in enormous costs for our health and social-care systems.

By gaining a better understanding of healthy musculoskeletal ageing and the risk factors associated with premature ageing and senescence, we can provide better care and develop new and better-targeted therapies for common musculoskeletal disorders. This review is the outcome of a two-day multidisciplinary, international workshop sponsored by the Institute of Advanced Studies entitled “Musculoskeletal Health in the 21st Century” and held at the University of Surrey from 30th June-1st July 2015.

The aim of this narrative review is to summarise current knowledge of musculoskeletal health, ageing and disease and highlight strategies for prevention and reducing the impact of common musculoskeletal diseases.

## Background

**Fig. 1:**
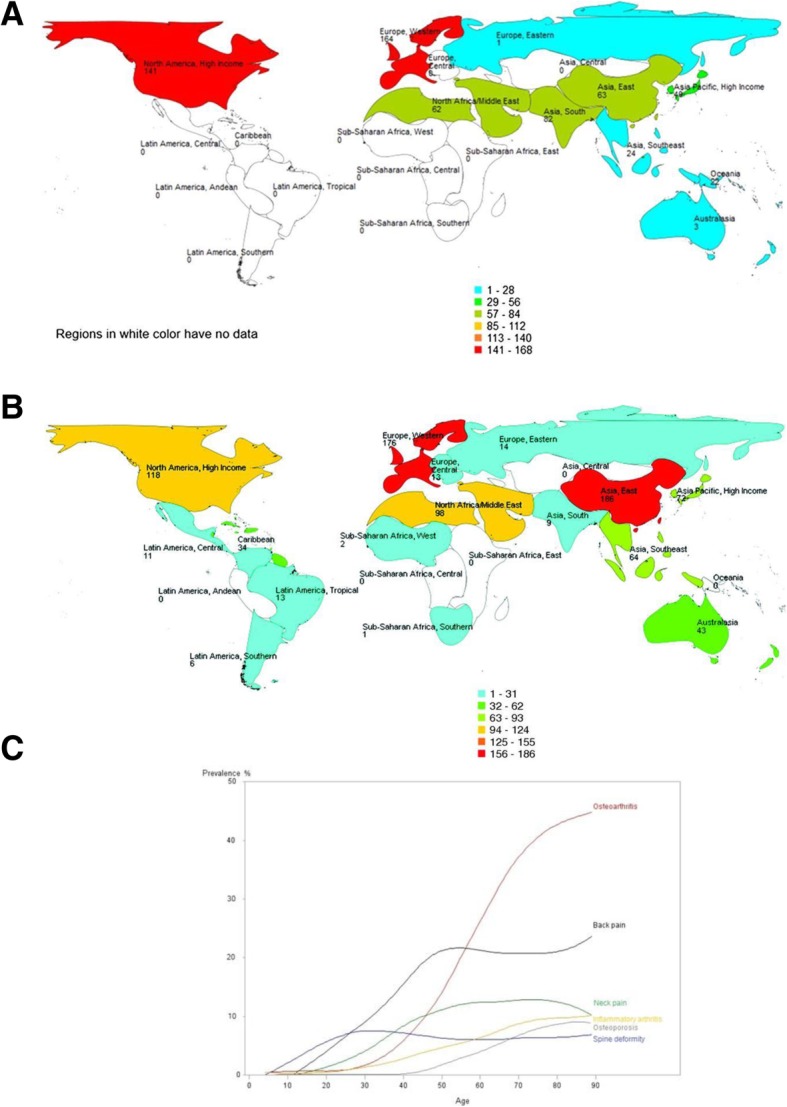
**a** The global burden of hip and knee osteoarthritis; estimates from the Global Burden of Disease Study 2010. The numbers show the number of case studies reported in the literature for each country, extracted via a systematic review process. Reproduced from Cross M, Smith E, Hoy D, et al. The global burden of hip and knee osteoarthritis: estimates from the Global Burden of Disease 2010 study. Note the absence of data from Central America, South America and Sub-Saharan Africa. Annals of the Rheumatic Diseases Published Online First: 19 February 2014. doi: 10.1136/annrheumdis-2013-204,763. **b**. The global burden of musculoskeletal disease attributable to low bone mineral density. The numbers show the number of case studies reported in the literature for each country, extracted via a systematic review process. Reproduced from Sànchez-Riera L, Carnahan E, Vos T, et al. The global burden attributable to low bone mineral density Annals of the Rheumatic Diseases Published Online First: 01 April 2014. doi: 10.1136/annrheumdis-2013-204,320. **c**. The prevalence rheumatic and musculoskeletal diseases in France. This figure highlights the dominance of osteoarthritis and back pain from the fifth to the 9th decade of life. Reproduced from Palazzo C, Ravaud JF, Papelard A, Ravaud P, Poiraudeau S (2014) The Burden of Musculoskeletal Conditions. PLOS ONE 9(3): e90633. 10.1371/journal.pone.0090633

**Fig. 2:**
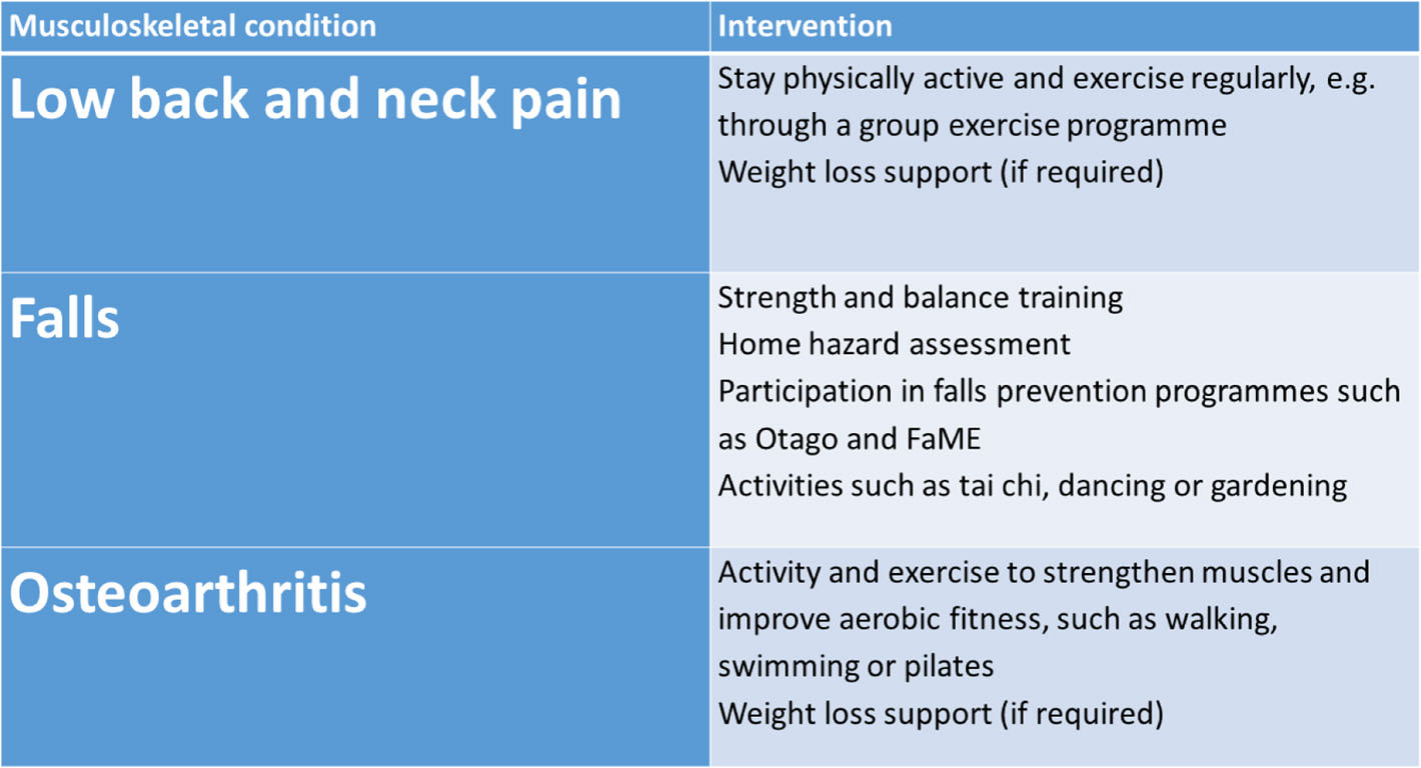
Evidence-based interventions for the musculoskeletal conditions that cause the most DALYs (Disability-adjusted life years) in England, including low back and neck pain, falls and osteoarthritis. https://publichealthmatters.blog.gov.uk/2016/01/11/preventing-musculoskeletal-disorders-has-wider-impacts-for-public-health/

The current rise in life-expectancy, the subsequent increase in numbers of older people and increasing pension costs have prompted the UK government to change policy, extend working years and delay pensionable age. By 2034 the number of people in the UK aged 85 and over is projected to be 2.5-times larger than in 2009, reaching 3.5 million and accounting for 5 % of the population. The number of people aged 65+ is expected to rise to over 16 million in the UK by the same time point (according to Age UK and the Office for National Statistics [1]). However, working for an extended period of time may not be feasible for those with major, or chronic, health problems. As careers are extended in different sectors, special attention will need to be paid to addressing the workplace needs of those with major health problems. According to the UK Department of Health and Age UK, 58% of those aged 60 and over report having a long-term condition, with 25% of over 60s having two or more health problems [1, 2]. This means that the majority of those aged 60 to retirement will already have multiple morbidities. Of these conditions, musculoskeletal diseases are considered to be one of the highest global burdens on individuals, health and social-care systems [3–6] (Fig. 1 a and b). It is important to note that although there is some epidemiological information about the burden of musculoskeletal disease in India [7, 8] and China [9] through COPCORD (Community oriented program from control of rheumatic diseases), equivalent, up-to-date and accurate epidemiological data does not exist for Central America, South America and Sub-Saharan Africa. Therefore, the true global burden of musculoskeletal disease is likely to be grossly underestimated. Common musculoskeletal conditions include osteoarthritis (OA), rheumatoid arthritis (RA), psoriatic arthritis (PsA), gout, lower back pain (LBP) and osteoporosis (OP). Between the fifth and ninth decade of life, OA and LBP are major contributors to musculoskeletal impairment (Fig. 1 c). Arthritis is of particular concern for the UK population as some 10 million people are now estimated to be suffering from the condition. Public Health England has published guidelines for evidence-based interventions to reduce the impact of LBP, falls and OA (Fig. 2). OA is the most common form of arthritis and the key risk factors are age, obesity, metabolic disease and prior joint injury. RA is an inflammatory joint disease with a strong genetic and immune basis that affects approximately 1% of the total global population. The incidence of OA and RA increases with age, as does LBP [10].

This narrative review is the outcome of a multidisciplinary, international workshop sponsored by the Institute of Advanced Studies (IAS) entitled: “Musculoskeletal Health in the 21^st^ Century” and held at the University of Surrey, UK, from 30th June-1st July 2015. The abstracts from this workshop were published in a special supplement of BMC Musculoskeletal Disorders in 2015. [11]. Diverse topics were discussed and debated, ranging from the global burden of OA to the link between diabetes and joint disease, diet and nutrition in arthritis, One Health [12], ageing, advances in imaging and musculoskeletal health and disease in military personnel and companion animals. One of the key deliverables of the international workshop was a paper that summarised the topics that were discussed. In this paper, we summarise and disseminate some of the key outcomes from the workshop in the context of currently available information, discuss the current challenges faced by society in relation to the rising burden of musculoskeletal disorders and review existing strategies and recommendations for prevention and mitigation of the impact of these diseases.

The workshop reached an important consensus statement, namely that development of more precise diagnostic and prognostic tools and targeted treatments is necessary, along with better disseminated preventative measures, which is in complete alignment with those outlined by the Arthritis Research UK (now rebranded as Versus Arthritis) approach plan (2014). In order to achieve this, greater transfer and translation of knowledge between the veterinary and human fields is needed as well as more funding for musculoskeletal research and investigation of populations with rare diseases and those with higher incidences of joint injuries, such as youth sport and the military.

## Current societal challenges

### Physical inactivity and sedentary behaviour

Sedentary behaviour and the rapid growth of legions of video game, social media and movie-streaming addicted couch potatoes is a consequence of sustained and systemic urbanisation in developed countries, including the United States, where nearly 50% of the population do not undertake even the bare minimum levels of aerobic activity recommended by Physical Activity Guidelines [13]. This rise in sedentary behaviour is seen as a major risk factor for a number of chronic diseases recognised by the National Health Service (NHS), costing around £1billion a year in the UK, and is recognised as a substantial global economic burden [14]. Statistics recently provided by the Centre for Economics and Business Research show the cost of “doing nothing”: half a million Europeans die every year as a result of being physically inactive and this costs the economy over €80bn annually [15]. This global challenge requires urgent and feasible solutions. Increasing physical activity and optimising exercise (as recommended by Arthritis Research UK/Versus Arthritis and World Health Organisation (WHO)) is seen as an optimal way to improve musculoskeletal health. Only 36% of the adult population in the UK take part in moderate intensity physical activity of about 30 min at least once a week [16]. An increasing body of evidence is showing that even the effects of a sedentary lifestyle (for example, of those with a desk job) can be mitigated by a small amount of activity every day. A recent meta-analysis of the data from studies involving over 1 million individuals concluded that an hour of moderate level activity per day eliminated the increased risk of death associated with 8 h of sitting [17]. Interestingly, the study also found that these levels of activity did not have an effect on the increased risk of death associated with high levels of leisure-time sedentary behaviour such as watching television. Whilst this level of activity is far more than those recommended by WHO, it is clear that keeping a moderate level of physical activity is a key requirement for healthy ageing and maintaining musculoskeletal health [17]. WHO recommendations suggest that healthy individuals should take around two hours a week of moderate physical activity or approximately 20 min a day of doing any kind of physical activity like brisk walking. This level of exercise, which involves elevation of heart-rate, has been associated with lower lifetime risk of cardiovascular disease in a 25 year longitudinal study of approximately 13,000 adults [18]. In addition to benefits for musculoskeletal health, improvements in cardiorespiratory fitness can be achieved by changing sedentary behaviour to achieve a low-intensity physical activity such as walking [19]. Furthermore, in patients with knee OA, improvement of locomotor function, including balance and strength, and a reduction in pain was seen following supplementation of home exercise with an eight-week class-based programme [20]. Patients suffering with chronic LBP who were given an exercise programme combining muscle strength, flexibility and aerobic fitness also reported a reduction in stiffness, which can result in back pain [21]. The idea of exercise for rehabilitation of musculoskeletal injuries has been widely accepted for many years now, and the idea of prescribing exercise as a preventative health measure is also more widely investigated, with guidelines around the type, frequency and duration of activity being considered [22].

Sedentary behaviour is also contributing to the rise in obesity and type-2 diabetes. Obesity is a major contributor to the development and progression of OA [23] and numerous epidemiological studies have confirmed the link between adiposity and joint degeneration. The prevalence of diabetes mellitus is between 7 and 14% globally [24]. Diabetes is an important predictor for severe forms of arthritis [25] and has recently been shown to be an independent risk factor for the progression of OA in men [26]. This shows that measures to increase levels of physical activity will not only increase musculoskeletal (MSK) health but also decrease the risk of suffering from obesity-related diseases such as diabetes. This will reduce the numbers in the population susceptible to MSK disorders and ill health.

### Healthy ageing and physical exercise

Whilst ageing is inevitable, the benefits of exercise on the ageing body are numerous and, in some circumstances, can reduce the manifestations of ageing, particularly the "ageing phenotype" of the elderly. A recent systematic review looked at evidence supporting nutrition and physical activity in the prevention and treatment of sarcopenia [27]. Sarcopenia results in loss of muscle strength and mass and this can lead to weakening of musculoskeletal structures and impair tendon, ligament, bone and cartilage function, which will destabilise the joint and increase the risk of arthritis and other musculoskeletal disorders. The authors identified a total of 37 randomised control trials to explore the effect of combined exercise and nutritional intervention for overcoming muscle sarcopenia. They concluded that physical exercise has a positive impact on muscle mass and function in healthy subjects aged 60 and above, however, there were huge variations in outcomes connected with dietary supplementation, highlighting the difficulties associated with cohort studies of well-nourished human beings, where the interactive effects of dietary supplementation may be masked or completely limited [27]. Physical exercise improves muscle performance by increasing the ratio of type I to type II muscle fibres and increasing the cross-sectional area of type II muscle fibres [28].

A European Society for Clinical and Economic Aspects of Osteoporosis, Osteoarthritis and Musculoskeletal Diseases (ESCEO) taskforce looked at dietary protein and vitamin D and calcium supplementation and recommended higher protein intake in combination with physical exercise particularly in post-menopausal women at risk of developing menopause-associated musculoskeletal disease, such as OP [29]. Physical exercise programmes improve strength and balance in ageing women with OP [30]. Fragility fracture risk, associated with OP, can be decreased by following an exercise programme, as exercise increases bone density and reduces inflammatory markers [31]. However, the incidence of OP is usually highest in elderly females who are most unlikely to perform the dynamic exercises needed for bone modelling/remodelling [32]. This highlights the challenge of preventing OP and OP-related fractures in elderly females that cannot perform ballistic exercise. Some medications are available for these frail patients but having an active lifestyle from an early age and following recommendations for exercise could be more beneficial. The same principle applies to frailty in ageing companion animals, where life-long spontaneous exercise significantly slows down the progression of frailty [33].

Physical activity is also known to increase insulin mediated glucose uptake and, in individuals without type-2 diabetes, exercise positively impacts on systemic glucose homeostasis. However, in patients with type-2 diabetes where beta-cell impairment is significant, physical training does not decrease insulin secretion [34]. A recent study found that a 30 min daily increment in moderate to vigorous intensity physical activity also significantly reduced glycated haemoglobin, a measure of type-2 diabetes risk [19]. Physical exercise reduces the risk of cardiovascular and metabolic comorbidities associated with joint diseases.

In addition to the positive effects of exercise on physical and mental well-being, there is also ample evidence to suggest that exercise and mechanical loading have a positive impact at the molecular, cellular and tissue levels. For example, in tendons, where ageing decreases the potential for cell proliferation and number of stem/progenitor-like cells, it has been shown that exercise/loading can induce an increase in tendon collagen synthesis [35], increasing tendon strength. In an animal model of ageing, it was found that moderate exercise could also enhance the quality of tissue produced during healing of injured tendons [36]. There is plenty of published evidence to support a positive role for physical exercise and mechanical loading for cartilage [37] and bone health [38].

### Obesity

The rise in sedentary behaviour and unhealthy diets contributes to the global obesity epidemic and the sharp rise in the incidence of type-2 diabetes. When combined these are powerful risk factors for cardiovascular and neurodegenerative diseases, which further complicates the management of musculoskeletal diseases. As with physical inactivity, the NHS now recognises obesity as an independent major risk factor for population ill-health, costing the UK over £5billion. Obesity is thought to be a key co-morbidity of many musculoskeletal conditions and is closely related to the development of OA, one of the commonest musculoskeletal health issues. Reducing levels of obesity in the adult population may lead to reduced occurrence of OA and can alleviate some of the pain of the condition. Obesity also happens to be one of the most modifiable risk factors for OA [39]. Exercise and weight loss has been successfully trialled in overweight and obese adults with knee OA [40]. There are ongoing trials assessing the effects of combined physical activity and weight loss and it will be interesting to see how combining exercise and weight loss may act synergistically to improve musculoskeletal function. It is now clear that combining modest weight loss with moderate exercise can provide the best overall improvement in symptoms of pain and joint function. Furthermore, improved diet, moderate exercise and weight loss are probably the best short-term solutions for the ineffective surgical interventions for OA patients.

### Childhood obesity and physical inactivity

Reducing obesity in children may also reduce the risk of developing musculoskeletal pain later in life [41], although the full impact of obesity on development of the child’s musculoskeletal system is still poorly understood [42]. A major contributor to the obesity epidemic is the lack of physical exercise in the population, with many people leading an increasingly sedentary lifestyle. Similarly, unhealthy diets are a common problem, due to the ready availability of a bewildering variety and quantity of fast foods, ready meals and the relentless advertising of these products. The low nutritional value and high calorie content of these foods further contributes to the obesity epidemic. They may also play a role in the high prevalence of type-2 diabetes and cardiovascular diseases which are exacerbated by a lack of fitness and by inactivity.

### The companion animal link

The health issues that impact on society are not just limited to humans. We co-exist with a variety of companion animals and share the same diet and environment. In a study of approximately 700 dogs, 40% were overweight and 20% obese [43]. In addition, there is a strong correlation between the canine obesity and the BMI of their owners, indicating that the lifestyle and diet of the owners is having a direct impact on their pets as they share the same environment, and probably with similar impacts on their families [44]. This highlights an opportunity for vets and medics to collaborate to tackle obesity, diabetes and cardiovascular co-morbidities that impact on humans and their companion animals.

### Dietary factors

It is generally accepted that maintaining a healthy weight can help to improve musculoskeletal health and prevent degenerative diseases, but research also focuses on whether dietary factors can influence disease progression. Eating a varied diet high in fresh fruit and vegetables is recommended by many health organisations. A combined regime of exercise and increased intake of fruit and vegetables increased life expectancy in women; those in their 70s with the highest level of activity and vegetable consumption were eight-times more likely to survive a five-year follow up period [45]. However, few direct links between fruit and vegetable intake and improved musculoskeletal health have been shown but one three year follow-up study of nearly 400 adults showed that diets high in potassium (from fruit and vegetable intake) reduced the amount of muscle loss in adults > 65 years [46]. Dietary flavonoid intake, the compound found in many fruit and vegetables, was positively correlated with good bone health (measured by bone mineral density and bone resorption) in a population of peri-menopausal women [47].

As current treatment options in OA are very limited, OA patients may benefit from the ability to self-manage their condition through improving their diet [48]. Vitamin D, calcium and protein (particularly protein) optimise muscle, bone and functional outcomes in older people reducing falls and fractures [49]. Calcium and protein intake work together to optimise bone health [50]. Previously, it was thought that, in older patients, diets too high in protein should be avoided due to the detrimental effect on the kidneys. However, increasing evidence now shows that protein levels should not be decreased, as the effects of metabolic acidosis on the kidneys can be offset by increased fruit and vegetable intake (as these foods decrease renal acid load) [51, 52].

Several studies recommend the benefits of supplementing the diet with “nutraceuticals”. A recent systematic review found promising but nevertheless limited research evidence to support the oral use of several herbal supplements including *Boswellia serrata* extract and pycnogenol, curcumin and methylsulfonylmethane in people with OA despite the poor quality of the published studies [53]. Dietary strategies for improving musculoskeletal health can include consumption of long-chain fatty acids and vitamins D and K [54, 55] as well as decreasing blood cholesterol [56]. OA patients should ensure that they meet the recommended intakes for micronutrients such as vitamin K, which has a role in bone and cartilage mineralization. However, the currently available evidence for a role of vitamin D supplementation in OA is unconvincing [48]. Research has focussed on a number of dietary supplements to modulate progression of the disease or ease joint stiffness (and therefore pain). A diet rich in antioxidants may provide a useful therapeutic tool for athletes, improving tissue repair, although the optimum dosage is unknown [57]. Combining exercise with a dietary supplement of whey protein fortified with vitamin D is effective at increasing muscle mass and strength in elderly people affected with sarcopenia [58].

Both human and veterinary research has shown promise for a number of other natural products or compounds derived from natural sources for alleviation of arthritic symptoms. Green-lipped mussel extract has been shown to be an effective chondroprotective agent [59], reducing OA symptoms in dogs with OA [60]. More recently, fish oil and krill oil have been found to have even greater protective effects against proteoglycan and collagen degradation in an in vitro model of canine arthritis [61]. These substances are all known to be rich in long-chain omega-3 polyunsaturated fatty acids. A separate study found that supplementing the diet of dogs with eicosapentaenoic acid and docosahexaenoic acid (both long-chain omega-3 polyunsaturated fatty acids) significantly reduced the clinical signs of OA [62]. In humans, these substances have also been demonstrated to provide an alternative to anti-inflammatory pain medications for the relief of chronic neck and back pain as well as joint pain in rheumatoid arthritis [63, 64]. Whilst the molecular mechanism of action of these fatty acids is not entirely understood, it has been shown that these compounds reduce the expression of cartilage-degrading proteases and inflammatory cytokines [65].

Curcumin is a well-known plant-derived compound with anti-oxidant and anti-inflammatory properties. Its effects have been described in numerous chronic illnesses in humans, including diabetes, allergies and arthritis. It modulates growth factors, transcription factors and inflammatory cytokines [66]. Interestingly, a recent systematic review, has investigated the efficacy and safety of dietary supplements for OA human patients and found that lesser known supplements such as curcumin from *Curcuma longa* and Boswellic acid from *Boswellia serrata* were more effective than well-known nutraceuticals, such as glucosamine and chondroitin [67]. Glucosamine and chondroitin are popular supplements that have been suggested to promote healthy joint function. However, there is little evidence of their benefit [67]. Glucosamine was recommended in early guidelines issued by the European League Against Rheumatism (EULAR) and the Osteoarthritis Research Society International (OARSI) for the management of knee OA [68, 69] but it was not recommended in the National Institute for Health and Care Excellence (NICE) guidelines and in the most recent set of OARSI recommendations it was identified as being “uncertain” [70]. More recent guidelines published by EULAR, OARSI and ACR do not recommend glucosamine.

In vitro evidence for glucosamine is generally good. At relatively high concentrations, glucosamine has been shown to have a protective effect on cartilage and synovial fluid; however many clinical trials have shown that this substance is unable to reach the tissue that it is meant to affect in appropriate and sufficient doses [68]. Structural heterogeneity of chondroitin sulphate, another supplement commonly taken alongside glucosamine, means that it is difficult to see consistent effects of this supplement, and the supplements available on the market are unlikely to be pure due to contamination with other glycosaminoglycans during the manufacturing process [71]. There is some evidence that glucosamine and chondroitin sulphate together provide superior effects on the inhibition of nitric oxide and prostaglandin synthesis and on protection of cartilage structure, than when applied alone [72, 73]. This combination may also be effective with the addition of manganese ascorbate, as shown in patients with knee OA [74]. However, this is again in an in vitro model, so it is doubtful that these substances would reach the required tissues at an appropriate dose. A small number of studies have found side effects of glucosamine and chondroitin sulphate treatment, which include inducing insulin resistance and glucose metabolism disorders [75]. There is also concern over the formulation of glucosamine, as it is commonly given as a salt (glucosamine sulphate potassium/sodium chloride) which could affect renal function, particularly in elderly patients, who are arguably more likely to be taking the supplement [68].

The gut microbiome is also an area of increasing focus for health research. There is an association between The Western Ontario and McMaster Universities Osteoarthritis Index (WOMAC) pain scores of hip and knee OA and the gut microbiome of individuals [76], so possible future dietary interventions for OA could include maintaining a healthy gut flora. Since dysbiosis of the intestinal microbiota is strongly associated with the pathogenesis of several metabolic and inflammatory diseases, it is conceivable that also the pathogenesis of OA might be related to it. However, the mechanisms and the contribution of intestinal microbiota metabolites to OA pathogenesis are still not clear [77]. Other foods and food supplements, such as blueberry leaves and milk thistle, have been found to have an anti-inflammatory or anti-oxidant effect in other body systems, however the effect of their function on the musculoskeletal system is yet to be realised [78, 79].

### Other co-morbidities and musculoskeletal disorders

There is increasing awareness of the effects of the pain from musculoskeletal disorders on mental health. A study on the effects of musculoskeletal chronic pain of 5900 individuals (including fibromyalgia or chronic back or neck pain) indicated that they are at increased risk of poor mental health and diminished quality of life compared to those who did not report suffering from these conditions [80]. Whilst the links between mental health and musculoskeletal disorders are complex, it is thought that living with the pain of OA can lead to depression and anxiety; conversely, psychological distress and depression worsen pain [81–83]. This can develop into a vicious cycle with worsening pain and low mood. An Australian study has found a strong association between musculoskeletal health and mental health; 470,000 more Australians had both a musculoskeletal condition and a mental disorder than would be expected if occurrences of the two conditions were independent of each other [84]. Chronic insomnia can also indicate musculoskeletal pain, as the two commonly co-occur, and doctors should enquire about sleep patterns in patients consulting with pain conditions [85].

A significant number of people suffering from OA also have OP, which affects approximately 3 million people in the UK. However, it was found that unless bone mineral density measurements were taken from sites other than the OA affected joints, there was a high likelihood of an osteoporosis diagnosis being missed [86]. Another musculoskeletal disorder, fibromyalgia, is often associated with chronic fatigue, sleep disorders, irritable bowel syndrome and other psychological disorders, as well as cardiovascular dysregulation [87].

Chronic musculoskeletal disorders can also aggravate other disease conditions, due to their activity-limiting effects. Where patients are diagnosed with a musculoskeletal condition, it often means that they will have limited mobility or that activity is painful to them. This restriction of movement can then cause other ill health, such as obesity or diabetes, or contribute to the effects of respiratory disease [88]. For example, in a population of war veterans, arthritis was shown to be associated with diabetes, obesity and cardiovascular comorbidities [89]. Obesity has also been shown to reduce the efficacy of anti-TNF treatment in rheumatoid arthritis [90].

## Diagnostic and prognostic tools for understanding mechanisms of disease

Biomarkers are routinely being used for diagnosis of various diseases. These can include imaging techniques, as well as detection and measurement of biochemical markers found in the blood and urine. Currently most OA diagnoses are made by radiography, once the patient has presented with severe joint pain, by which point there is little the clinician can do except manage that pain at some level. Biomarkers for early diagnosis of diseases could help to detect at-risk individuals and they could then be put onto treatment plans to help prevent further development of disease. Currently diagnosis of OA, for example, is generally confirmed using imaging techniques such as radiography [91]. Ultrasound is commonly used for diagnosis of soft tissue disorders such as myopathies [92]. Magnetic Resonance Imaging (MRI) and Computed Tomography (CT) can also be useful imaging techniques, however these more expensive methods are less frequently used. MRI is the only one of these techniques that is capable of assessing all the structures of the joint, including cartilage and ligaments, in 3D [91]. Radiography and CT techniques can be limited, however, as the degree of joint damage seen does not necessarily correlate with the pain the individual is experiencing, or the actual degree of cartilage damage, for example. Radiographic diagnosis of arthritis can be determined by joint space width measurements and osteophyte development [93]. Ultrasound can also provide a useful view of the different tissues within a joint, as it can provide a view on early inflammatory features and allows for detailed measurements of the joint structures [94]. Development of more sophisticated imaging techniques and image analysis tools is necessary to correctly diagnose stages of musculoskeletal diseases. While some techniques can distinguish small changes in joint tissues during disease, it is unclear how these correlate to specific grades of musculoskeletal conditions [95]. A technique that is starting to receive more attention is the use of non-invasive probes for monitoring joint damage and inflammation. Radiotracers, or contrast agents, are used for detecting inflammation to provide a method for detection of specific subtypes of inflammatory activity in musculoskeletal conditions, by providing earlier and more reliable assessments of tissue inflammation  [96].

Gait analysis can be used as a biofeedback marker of musculoskeletal health as it provides physical functional outcome measures to quantify improvement, monitor treatment and can provide early diagnosis of mechanical compensation due to patient pain or discomfort. Diverse gait-analysis technologies have been developed and used in humans and sport horses over the past 40 years, and are constantly being refined to provide reliable measures of improvement from disease [97]. Current technologies include wearables, inertial measurement units or accelerometers which are lightweight, wireless devices to investigate activity levels, gait patterns and fitness parameters for humans and other animals [98–100]. Real-time or delayed-time parameters that can be analysed include ground reaction forces and foot-pressure distribution, the kinematics of joints and segments, along with dynamic electromyograms [101]. The information gained from these analyses enables clinicians to quantify, and therefore monitor and evaluate, gait and posture parameters such as asymmetry and other abnormal movement patterns, possibly indicating pain or discomfort in the musculoskeletal system. Increasing evidence indicates that cytokines and mediators together with mechanical stress are key to the development of cartilage damage; this mechanical stress due to abnormal movement patterns can also be quantified by gait analyses [37]. Gait analysis has great clinical value as a test for patients with neurological and orthopaedic disorders as it provides quantifiable, objective, data to aid the clinician in selection of any surgical procedure needed and then to monitor outcomes and follow up post-surgery. This is a valuable addition to the use of traditional clinical examination. Nowadays, more accurate and user-friendly technology for gait analysis allows investigation of musculoskeletal diseases in human patients [101] and other species, with the goal of obtaining a better definition of specific clinical hallmarks of diseases such as rheumatoid arthritis [102] and OA [103–105].

Imaging tools, diagnostic biomarkers and gait analysis should be combined for a more integrated approach to diagnosis of musculoskeletal disorders to ensure that the clinician not only has a clear and quantifiable overview of the patient’s clinical signs, but is also able to consider what is happening at a molecular and tissue level.

## Management of musculoskeletal diseases

Common musculoskeletal disorders include LBP, fibromyalgia, gout, OA, tendinitis and RA. These result in pain and disability, affecting quality of life and productivity. To address this, Arthritis Research UK, recently rebranded as Versus Arthritis in the UK, has developed several recommendations to improve musculoskeletal health, including advice on diet and lifestyle as preventative measures [41]. However, none of these recommendations is related to actual treatment of disease or improvement of diagnostic tools. Treatment of OA, for example, currently only consists of pain management, which is often insufficient, whilst diagnosis is generally limited to clinical examination by a general practitioner or, in some cases, diagnostic imaging.

Management of musculoskeletal diseases should start with proper and complete pain management, including accurate diagnosis and grading of pain [106]. As discussed previously, pain from musculoskeletal conditions severely affects patients’ quality of life. In humans, paracetamol and non-steroidal anti-inflammatory drugs (NSAIDs) are most often prescribed to treat OA pain, with some clinicians preferring paracetamol/opioid combinations or an opioid alone, depending on patient age and other comorbidities (for example, renal disease, diabetes, hypertension, gastrointestinal, etc.) [106]. These medications may be appropriate for the majority of patients, however, there are some barriers to optimal pain management, including patient compliance, self-medication and lack of monitoring by the clinician [106]. It is recognised that opioid misuse in the U.S. has reached epidemic proportions [107], for which the U.S. Department for Health and Human Services has announced a new combative strategy [108]. Therefore, other treatment options may need to be considered if the pain cannot be appropriately controlled or if the prescription of particular medications is problematic in that country.

## One health, OA and learning from studies in the canine species

OA is also a commonly presented condition in dogs. The recommended conservative management in this species usually includes nutritional management and weight control (with considered exercise options), alongside pain management/disease modifying agents and physical rehabilitation [109]. The usual approach when a canine presents with OA is to prioritise weight loss and exercise, as the canine patients are often overweight or obese, thus weight management is seen as a priority in these cases [110]. Similarly, it is becoming increasingly recognised that obesity is a risk factor for OA in humans, with increasing prevalence in the last few decades [111, 112]. Where canine pain is managed, the usual treatments are similar to human medicine and include opioids and NSAIDs. Systemic and intraarticular corticosteroids have been shown to be effective for pain management in dogs and horse, despite being less commonly used in these species, and may provide a protective articular effect [113, 114]. In contrast, intraarticular corticosteroid injections for human knee osteoarthritis have been shown to give similar reductions in pain as the placebo injection [115]. The latter could be explained by a mechanical effect of the fluid volume being injected. Research into musculoskeletal conditions in veterinary species is lacking in quantitative markers of evaluation, such as objective gait analysis. Despite lameness scoring by a veterinarian (a semi-quantitative tool), owner perception is often relied upon to determine patient improvement. The “caregiver placebo effect” is reported on in veterinary literature, affecting both owner’s and veterinary practitioner’s judgement when they are assessing an animal’s lameness against an objective measure [116]. Further research into quantitative biomechanical markers in veterinary species is essential, along with increased use of objective measures in clinical practice.

In humans and veterinary species, the treatment regimens are very similar with corticosteroids. The usual dosing pattern would be one intraarticular injection every six weeks, with no more than 3–4 per year. Long-term steroid use (i.e. more often than four times per year) is recommended to be avoided in all species due to systemic side-effects [117]. NSAIDs are also known for having side effects in the gastrointestinal system, with potentially severe effects in the lower gastrointestinal tract, as well as in other systems [118]. Therefore, finding alternatives to these therapies for high-risk patients seems to be the logical option. Some possibilities may be topical NSAIDs, which have fewer side effects [119] or other modalities such as photobiomodulation (low level laser therapy; LLLT) [120]. Animal studies suggest that at the right dose, laser therapy can be more effective at modulating the inflammatory process, than topical NSAIDs which treat pain alone [120]. Treatment of the inflammatory processes may also decrease the pain that is reported with many musculoskeletal disorders or injuries. Traditionally, people may use cryotherapy or topical NSAIDs such as diclofenac for treatment of acute musculoskeletal injuries. However, a recent study showed that LLLT was more effective at decreasing pro-inflammatory cytokine release in an animal model than topical diclofenac and cryotherapy [121]. Other treatments such as acupuncture, ultrasound and transcutaneous electrical nerve stimulation (TENS) are known to be effective for pain relief in both human and veterinary medicine, however, the methodological quality of some studies has been questioned and comparisons between the studies show large heterogeneity and significant publication bias [122]. For example, acupuncture has been shown to be effective for the short term relief of pain [123]; TENS was shown to be ineffective for musculoskeletal pain [124, 125]; ultrasound and shockwave therapy also do not appear to improve pain significantly [126, 127]. However, there do seem to be patient-reported beneficial effects of these treatments and reduced lameness in animals, which may mean that whilst there is no evidence for clinical mechanisms of action of these treatments, some of them may be working to improve patient health. Further, NSAIDs, opioids and topical steroids were found to be beneficial in the short term for pain relief, but not over a longer time period. In contrast, exercise therapy and psychosocial interventions not only relieved pain but improved function in a human primary care setting [122]. Psychosocial interventions may include self-management, behavioural and/or cognitive changes. These are longer-term therapies, which may improve patient outcomes as patients become empowered to manage their own conditions.

Physical rehabilitation will usually include several different techniques and modalities, in order to slow progression of the disease, improve patients’ activity levels and, in turn, decrease their level of disability. Some techniques used in rehabilitation include passive ranges of motion, where the therapist will move or manipulate the joint for the patient and thus increase the metabolism of the tissues. Rehabilitation can also include therapeutic exercises, which are controlled movements where the patient will perform active ranges of motion (i.e. the patient is self-motivated to move), achieving the same tissue metabolic and physical effects [128]. Rehabilitation in this manner aims to ensure muscle tone is improved and that joints are utilised effectively. Specifically, improving muscle tone should decrease the rate of progression of the disease (as the joint becomes stabilised) and hopefully decrease a patient’s pain levels. Whilst these therapies will not cure arthritis, improving the joint characteristics in this manner will enable the patient to keep active for longer.

Patients can be referred to a specialist for physical therapy and orthotics to improve posture and gait imbalances, which may not only prevent but improve musculoskeletal ailments that may ultimately result in injuries to cartilage and soft tissues. Identifying the needs of a patient before a musculoskeletal condition becomes an issue, for example, utilising workstation assessments (usually implemented by an Occupational Health professional, under employer guidance) in order to improve the ergonomics of items the patient uses every day and then training the patient in the best use of these items, will improve a patients’ posture and could lead to prevention of musculoskeletal injuries. Incorporating real-time visual feedback into the assessment of patient behaviour has been shown to be an effective tool for improving posture [129]. Increases in patient numbers referred for this preventative kind of therapy could reduce patient presentations at a later stage for more serious musculoskeletal complaints. This referral will only work if the general practitioner is able to identify these imbalances at an early stage.

It is clear that a holistic and individualised approach to managing treatment in musculoskeletal conditions is necessary, with the input of multidisciplinary health professionals, including general practitioners, dieticians, physical therapists and fitness specialists. Thus, the problem would need to be assessed from multiple viewpoints and at a much earlier stage with greater input from the patients and their commitment if behaviour change is required. Equally, this issue would also be more likely to fulfil patients’ needs and, most importantly, place greater emphasis on their medical background, lifestyle, any co-morbidities and their family history and susceptibility to developing musculoskeletal disease.

## Current research approaches for musculoskeletal diseases

**Fig. 3:**
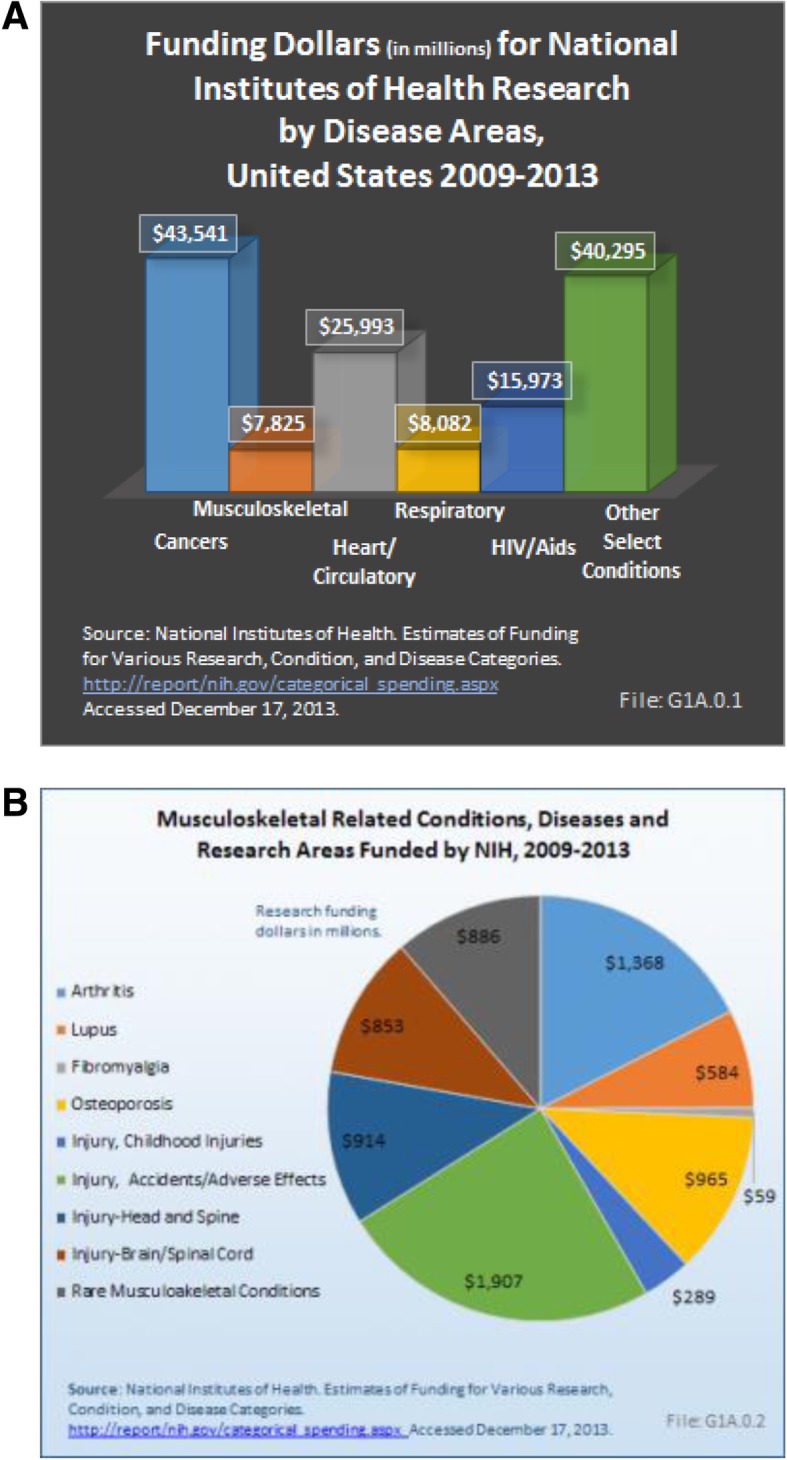
The burden of musculoskeletal disease in the United States and the funding gap for research on musculoskeletal conditions (2009–2013). **a**. Despite the major health care burden presented by musculoskeletal conditions, research funding falls well below that of most other conditions. Injury research accounted for half of the musculoskeletal condition research dollars ($4 billion) from NIH for the years 2009 to 2013. Funding for arthritis research is second, at $1.4 billion, followed by osteoporosis ($965 million). These numbers are well below the $8.6 to $55.2 billion in funding for the top 25 NIH research areas, dominated by cancer, cardiovascular disease and other disease areas. **b**. Research funding allocated to different musculoskeletal conditions by NIAMS. The data clearly indicate that musculoskeletal conditions receive less funding support compared to other disease areas despite the heavy burden on healthcare systems worldwide

Arthritis Research UK, recently rebranded as Versus Arthritis, has presented a new approach plan towards arthritis and related musculoskeletal conditions by providing a wealth of information to the public, funding and undertaking research, improving data collection and influencing related policies [41]. Whilst data on the levels of funding received for different conditions can be difficult to obtain for European countries, in the United States, musculoskeletal conditions received the least funding from the National Institutes of Health Research compared to other conditions such as cardiovascular disease or cancers (Fig. 3 a). Of those musculoskeletal conditions funded, the largest proportion went to investigating conditions resulting from injuries or accidents (Fig. 3 b).

Understanding common diseases such as OA and RA would help to develop better and more precise diagnostic and prognostic tools, improve treatment management and lead to the recommendation of effective preventive measures. There are currently few funding streams that enable researchers to join together and work on all these aspects of musculoskeletal health. A multidisciplinary approach combining these factors could not only improve patient diagnosis and outcomes but also help further our understanding of disease progression. Current research on musculoskeletal diseases is mainly focused on understanding the mechanisms of disease to identify a better and earlier diagnosis. At a cellular level this includes studies on chondrocytes and synovial tissue as well as osteoblasts [130–132]. Studies with animal models to investigate OA are being widely used to understand the disease and its symptoms such as pain [133–135]. Veterinary research is important, not only on established animal models but also on companion animals because species, such as rabbits, dogs and horses, are also commonly affected by similar musculoskeletal diseases with similar clinical signs to humans [136, 137]. As these animals have a shorter life span, this allows for observation of the complete process of disease over a shorter time period.

There is also the opportunity when studying rare musculoskeletal diseases, such as alkaptonuria (AKU), for researchers to determine whether treatments developed specifically for these can facilitate the development of new therapies for the more common disorders. There may also be common pathways in disease progression, which may help understanding of how and why individuals develop musculoskeletal problems [138].

In addition to the general population, there is also an increasing need for more research in musculoskeletal conditions affecting the military; in 2006 there were nearly three quarters of a million reported musculoskeletal injuries or conditions in the US military [139]. This sector of the population is an interesting target for research studies as it has a high incidence of musculoskeletal injuries. For example, it has been shown that conservative physical therapy is a valuable first approach for musculoskeletal conditions in a US naval department [140]. On the other hand, the vast majority of US army active soldiers are being prescribed NSAIDs, where more investigation is needed to stablish the pros and cons of this practice in that population [141]. These contradictions could be tackled by increasing the number of high-quality research studies and thus establish new guidelines and recommendations for the improved management of musculoskeletal health in the military.

Until the underlying mechanisms of these diseases are revealed and important details that give us more information are clarified, little can be done in terms of developing new treatment options. Although it is important to work on a preventative approach, treatment still needs to be optimised to reduce pain and disability, especially given the rising numbers of elderly people as well as an unfit, sedentary and overweight population.

## Conclusion

The burden of musculoskeletal diseases will only increase with an increasing ageing population and an increased number of people not taking diet, lifestyle, health and physical activity seriously. A pragmatic multidisciplinary approach to treatment and prevention is needed to tackle these problems; including raising public awareness of risk factors and improving the understanding of these approaches within the medical and scientific community. Behaviour change and patient participation is absolutely crucial for success. We must make use of efficient diagnostic tools available and develop new ones that enable earlier identification of MSK disease; and we need to investigate the mechanisms of disease progression that will lead to preventive measures and individually tailored and more holistic treatment programmes.

## Abbreviations

ACR: American College of Rheumatology
AKU: Alkaptonuria
COPCORD: Community oriented program for control of rheumatic diseases
CT: Computed Tomography
ESCEO: European Society for Clinical and Economic Aspects of Osteoporosis, Osteoarthritis and Musculoskeletal Diseases
EULAR: European League Against Rheumatism
IAS: Institute of Advanced Studies
LBP: Low back pain
LLLT: Low level laser therapy
MRI: Magnetic Resonance Imaging
NHS: National Health Service
NIAMS: National Institute of Arthritis and Musculoskeletal and Skin Diseases
NICE: National Institute for Health and Care Excellence
NIH: National Institutes of Health
NSAIDs: Non-steroidal anti-inflammatory drugs
OA: Osteoarthritis
OARSI: Osteoarthritis Research Society International
OP: Osteoporosis
PsA: Psoriatic arthritis
RA: Rheumatoid arthritis
TENS: Transcutaneous electrical nerve stimulation
WHO: World Health Organisation
WOMAC: The Western Ontario and McMaster Universities Osteoarthritis Index
